# What Is the Next Generation of Transcatheter Mitral Valve Repair Devices?

**DOI:** 10.3389/fcvm.2021.641691

**Published:** 2021-02-24

**Authors:** Mi Chen, Lizhong Sun

**Affiliations:** ^1^Department of Cardiac Surgery, Beijing Anzhen Hospital, Capital Medical University, Beijing, China; ^2^Department of Cardiac Surgery, University Hospital of Zurich, University of Zurich, Zurich, Switzerland

**Keywords:** transcatheter mitral valve repair, transcatheter mitral valve intervention, transcatheter device, mitral regurgitation, functional mitral regurgitation

## Abstract

In the evolving scenario of the transcatheter mitral valve repair (TMVr), TMVr devices constitute a rapidly expanding field. The standard classification includes edge-to-edge repair, direct annuloplasty, indirect annuloplasty, chordal/papillary muscular repair, and the others. However, the unknowns and uncertainties to innovate a high-performing device are addressed. In this viewpoint, the authors discuss the potential future of the next generation and the challenges of TMVr devices.

## Introduction

Mitral regurgitation (MR) affects 9.3% of people older than 75 years, while 2.5% for aortic stenosis (1). However, transcatheter mitral intervention devices have been developed to address an unmet clinical need for inoperable patients with symptomatic severe MR. Given the more catastrophic and less forgiving complications of transcatheter mitral valve replacement, transcatheter mitral valve repair (TMVr) may be associated with a superior safety profile. Various TMVr devices are classified based on the surgical technique (Table 1), including edge-to-edge repair, direct annuloplasty, indirect annuloplasty, chordal/papillary muscular repair, and the others (2–10). Transcatheter edge-to-edge repair devices are based on Alfieri surgical technique by anchoring the free edge of the mitral leaflets and produce a double orifice. Transcatheter direct annuloplasty devices obtain reduced mitral annular dimension by anchoring mitral annulus directly, whereas, transcatheter indirect annuloplasty devices accomplish repair through adherent anatomies, such as the coronary sinus (CS) and left ventricle. Acknowledging the lack of scientific evidence to date, it is difficult to predict what the ultimate future TMVr devices will be. The purpose of this viewpoint is to address the potential future scenarios considering four aspects: safety, learning curve, the variability of disease and anatomy, and long-term outcomes.

**Table 1:** Classification of transcatheter mitral valve repair devices.

	**Edge-to-edge repair**	**Direct annuloplasty**	**Indirect annuloplasty**	**Chordal/papillary muscular repair**	**Others**
Transfemoral	MitraClip^*^^†^^‡^	Cardioband^*^^†^	Carillon^*^^†^	Pipeline ^*^	
	Pascal^*^^†^	Mitralign^*^	Accucinch^*^	Mitral Butterfly	
		Millipede^*^		V-chordal	
Transapical	MitraFlex	Amend^*^	VenTouch^*^	Neochord DS 1000^*^^†^	Mitra-Spacer
			Mitraspan TASRA^*^	Harpoon neochords^*^	
				MitralStitch^*^	
Others		QuantumCor (RF)	MVRx^*^		Mitramaze
		Valfix	MitraLoop Cerclage^*^		
			Mitral Bridge		

* *In patients*.

† *CE mark approval*.

‡ *FDA approval*.

## Favorable Safety Profile

**Figure 1:**
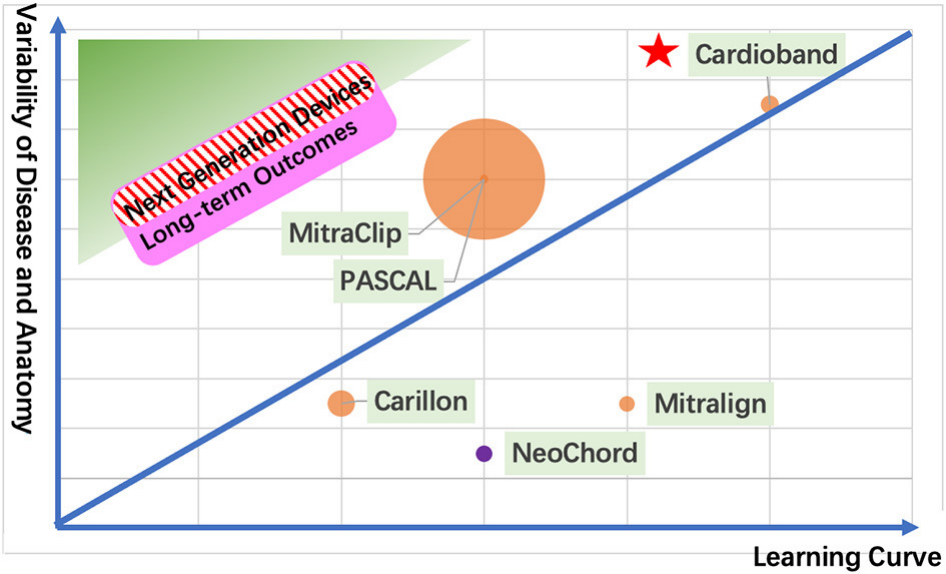
The next generation of TMVr devices. The green zone shows an ideal device with short learning curve and variability of disease and anatomy for transcatheter mitral valve repair. The pink zone represents a device with good long-term outcomes. The red stripe zone illustrates the next generation of devices. Each circle represents a CE mark approval TMVr device sized by its implant numbers (the orange circle represents the transfemoral approach; the purple circle represents the transapical approach). The red pentacle represents the surgical mitral valve repair as a reference.

Given the complexity and the heterogeneity of mitral valve anatomy and pathology, an excellent safety profile is mandatory as a permit to be available commercially. The safety of the device is first supported by its less invasive approach. It remains an increasing and strong interest to move from a transapical procedure toward a transfemoral and transeptal procedure shown by NeoChord DS 1000 (NeoChord, Inc., St. Louis Park, MN) via transapical approach and Pipeline (Gore Medical, USA) via transfemoral approach. Second, the less interference of the mitral valve apparatus and its adjacent anatomy, the safer it is. To date, the most common TMVr device is the MitraClip (Abbott, Abbott Park, IL, USA) with more than 100,000 implants based on the Alfieri edge-to-edge technique with only interference of mitral leaflets. COAPT registry illustrated high freedom from device-related complications of 96.6% (11). The Carillon (Cardiac Dimension Inc., Kirkland, WA, USA) is delivered through the CS to achieve indirect mitral annuloplasty with a relatively quick procedure. However, because of the contiguity of the circumflex artery with CS, 17% of cases due to coronary artery compression were reported in the TITAN II trial (12). Additionally, its safety to reproduce the surgical technique has been confirmed as the standard therapy. Cardioband system (Edwards Lifesciences, Irvine, CA, USA) is a surgical-like direct annuloplasty applied as an isolated or adjunctive approach, waiving the possibility of increasing the severity of MR. Meanwhile, because of the pure anatomical implant, Cardioband leaves the door open to further potential transcatheter mitral valve intervention (13). Finally, post-procedural safety matters. An ideal TMVr device does not require anticoagulation. A promising concept of transcatheter papillary muscular repair commenced, named Mitral Butterfly (AVVie, Vienna, Austria), but the thrombogenic potential of the device has not been studied clearly (14). Therefore, only CE marker–approved devices deemed with safety profiles are pooled in our further discussion (Table 2, Figure 1).

**Table 2:** CE mark approval devices.

**Device**	**Year of CE mark approval**	**No. of patients in trials**	**Variability of disease**	**Anatomical variations**	**Procedure duration**
MitraClip	2008	In total: 4,232	FMR DMR + + + +	+ + +	+ + +
PASCAL	2019	First-in-man: 23	FMR DMR + + + +	+ + +	+ + +
CardioBand	2015	Early feasibility study: 31 CE trial: 61	FMR + + +	+ + + + +	+ + + + +
Carillon	2009	AMADEUS: 48 TITAN: 36 TITAN II:36 REDUCE FMR:87	FMR + + +	+	+ +
Mitralign	2015	Early feasibility study: 71	FMR + + +	+	+ + + +
Neochord	2012	TACT:30 TOP-MINI:49	DMR +	+	+ + +

*+, + +, + + +, + + + +, + + + + + symbols represents the intensity from weak to strong*.

## Steeper Learning Curve

Burt et al. (15) reported that the learning curve determined the operative efficiency and long-term outcomes of surgical mitral valve repair. The impact of the operator and institutional experience can be more determinant in TMVr procedures, which were initially studied by Chhatriwalla et al. (16) in the review of the TCT Registry of 12,334 MitraClip procedures. Visual inflection points in the learning curves for acceptable procedural success were evident after ~50 cases, whereas optimal procedural success was observed in up to 200 cases. In the future, TMVr is to be considered as an alternative to surgery in eligible patients; operator experience may play a crucial role to achieve trace residual MR. With the gain of experience, the optimal procedural success and a wider spectrum of anatomical variations can increase not only in MitraClip but also in other TMVr techniques. A steep learning curve of the TMVr technique illustrates the outcomes to be less predictable in the early stage, which may prevent the promotion of the devices. Because of the lack of evidence of the learning curve for each TMVr device, the authors compared the procedure duration as the reference of the learning curve roughly (Table 2) (12, 17, 18).

## Variability Of Disease And Anatomical Variations

MitraClip was initially approved for organic MR and has subsequently been approved for functional MR (FMR) with a satisfactory variability of the disease. However, with the discordant clinical outcomes between COAPT and MITRA-FR trials, a notable concept of proportionate vs. disproportionate MR was applied to determine the feasibility of MitraClip, which means its variability of the disease becomes limited (11, 19). Although surgical mitral repair is the gold standard for severe degenerative MR (DMR), transcatheter edge-to-edge repair and transcatheter chordal implantation can be considered for inoperable patients with limited anatomical suitability. Concerning FMR with suboptimal surgical results and high perioperative mortality, transcatheter edge-to-edge repair and transcatheter annuloplasty play a promising and predominant role even with the lack of evidence showing the benefits of repair over replacement.

Another crucial issue is the suitability of anatomical variations of the TMVr devices, as PASCAL (Edwards Lifesciences, Irvine, CA, USA) was developed as a central spacer and optional independent grasping to increase the anatomical suitability (20). Meanwhile, the next-generation MitraClip G4 system provides four sizes with wide clips (NTW and XTW) and narrow clips (NT and XT) with an independently grasp ability to achieve a wider spectrum of anatomical variations (21). Maisano et al. (17) compared the feasibility of other TMVr devices, considering the anatomical reasons.

## Long-Term Outcomes

Even the oldest TMVr device, MitraClip, was implanted 17 years ago for the first time. Therefore, we are still far from having unbiased evidence to compare the long-term outcomes between the TMVr devices. We can seek every new TMVr device from three stages, 30-day, 1-year, and 5-year follow-up. (1) The 30-day follow-up demonstrates the safety and feasibility of the TMVr device. Maisano et al. (17) reported the 30-day outcomes of Cardioband. The full implant success, a significant reduction in the septolateral dimension in 29 of 31 patients, and no in-hospital mortality in 1-month follow-up demonstrated the safety and feasibility of percutaneous direct mitral annuloplasty. (2) The 1-year follow-up reveals the efficacy of the TMVr device. In COAPT and MITRA-FR trials, 1-year mortalities of MitraClip were 18.8 and 24.3%, respectively (11, 19), but the clinical outcomes are discordant when comparing with the controlled cohort. Nevertheless, the concept of proportionate vs. disproportionate FMR offered us a deeper perspective to estimate the efficacy of MitraClip. (3) The 5-year follow-up ensures the durability of the TMVr device. In 5-year outcomes of the EVEREST II trial, the rates of surgery for MV dysfunction between 1- and 5-year follow-up are comparably low in either TMVr or surgical therapy, whereas the MitraClip cohort more commonly required surgery for residual MR during the 1-year follow-up (22). These outcomes convinced us of the durability of MitraClip if it is a reliable implant during the first year. Only if a device is evaluated by the staged outcomes of 30-day, 1-year, and 5-year follow-up can a satisfactory spectrum of the safety and feasibility, efficacy, and durability ensure survival.

## Discussion

We provide an overview of four elements that should be taken into consideration for the next generation of TMVr devices (Table 2, Figure 1) and offer a comparison between the CE mark–approved devices. A safety profile always takes the first position for a new device. A shorter learning curve with a wider spectrum of disease and anatomy ensures the device's feasibility. Last but not least, satisfactory long-term outcomes make the TMVr device survive for a long time.

## Data Availability Statement

The original contributions presented in the study are included in the article/supplementary material, further inquiries can be directed to the corresponding author.

## Author Contributions

MC: concept, design, clinical studies, and manuscript preparation. LS: manuscript review.

## Conflict of Interest

The authors declare that the research was conducted in the absence of any commercial or financial relationships that could be construed as a potential conflict of interest.

## Abbreviations

TMVr: transcatheter mitral valve repair
MR: mitral regurgitation
DMR: degenerative mitral regurgitation
FMR: functional mitral regurgitation.
